# A single dose of noradrenergic/serotonergic reuptake inhibitors combined with an antimuscarinic does not improve obstructive sleep apnoea severity

**DOI:** 10.14814/phy2.15440

**Published:** 2022-08-27

**Authors:** Luke D. J. Thomson, Shane A. Landry, Simon A. Joosten, Dwayne L. Mann, Ai‐Ming Wong, Tim Cheung, Mulki Adam, Caroline J. Beatty, Garun S. Hamilton, Bradley A. Edwards

**Affiliations:** ^1^ Department of Physiology, School of Biomedical Sciences and Biomedical Discovery Institute Monash University Melbourne Victoria Australia; ^2^ Turner Institute for Brain and Mental Health Monash University Melbourne Victoria Australia; ^3^ School of Clinical Sciences Monash University Melbourne Victoria Australia; ^4^ Monash Lung, Sleep, Allergy and Immunology Monash Health Melbourne Victoria Australia; ^5^ Monash Partners – Epworth Melbourne Victoria Australia; ^6^ School of Information Technology and Electrical Engineering The University of Queensland Brisbane Queensland Australia

**Keywords:** combination therapy, muscarinic antagonists, noradrenergic, obstructive sleep apnoea., pharmacotherapy, serotonergic, sleep disordered breathing, upper airway

## Abstract

Previous trials have demonstrated that the combination of noradrenergic reuptake inhibitors with an antimuscarinic can substantially reduce the apnoea‐hypopnoea index (AHI) and improve airway collapsibility in patients with obstructive sleep apnoea (OSA). However, some studies have shown that when administered individually, neither noradrenergic or serotonergic agents have been effective at alleviating OSA. This raises the possibility that serotonergic agents (like noradrenergic agents) may also need to be delivered in combination to be efficacious. Therefore, we investigated the effect of an antimuscarinic (oxybutynin) on OSA severity when administered with either duloxetine or milnacipran, two dual noradrenergic/serotonergic reuptake inhibiters. A randomized, double‐blind, 4 way cross‐over, placebo‐controlled trial in ten OSA patients was performed. Patients received each drug condition separately across four overnight in‐lab polysomnography (PSG) studies ~1‐week apart. The primary outcome measure was the AHI. In addition, the four key OSA endotypes (collapsibility, muscle compensation, arousal threshold, loop gain) were measured non‐invasively from the PSGs using validated techniques. There was no significant effect of either drug combinations on reducing the total AHI or improving any of the key OSA endotypes. However, duloxetine+oxybutynin did significantly increase the fraction of hypopnoeas to apnoeas (*F*_Hypopnoea_) compared to placebo (*p* = 0.02; *d* = 0.54). In addition, duloxetine+oxybutynin reduced time in REM sleep (*p* = 0.009; *d* = 1.03) which was positively associated with a reduction in the total AHI (*R* ^2^ = 0.62; *p* = 0.02). Neither drug combination significantly improved OSA severity or modified the key OSA endotypes when administered as a single dose to unselected OSA patients.

## Graphical abstract

Milnacipran or duloxetine when combined with oxybutynin does not improve obstructive sleep apnoea severity when assessed using the AHI" cd_value_code="text

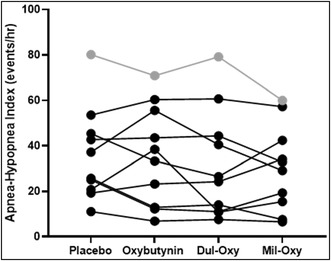

## 1 INTRODUCTION

Sleep‐induced hypotonia of upper airway muscles can induce collapse of the upper airway leading to obstructive sleep apnoea (OSA); a chronic and serious health condition affecting an estimated 1 billion people worldwide (Benjafield et al., 2019). Mechanistic studies have shown that withdrawal of noradrenergic and serotonergic activity during sleep is responsible for decreased upper‐airway muscle activity and subsequent increased airway collapsibility during NREM sleep (Kubin, 2016). Therefore, targeting these neurochemical mechanisms with pharmacological agents has been trialed as a treatment strategy for OSA.

Evidence from animal data and human trials have shown that norepinephrine is predominantly responsible for increasing genioglossus tone (the main pharyngeal dilator muscle; Fenik et al., 2005; Taranto‐Montemurro, Edwards, et al., 2016). However, human trials using noradrenergic agents, such as desipramine or atomoxetine in isolation have had minimal effect on OSA severity at a group level (Bart Sangal et al., 2008; Taranto‐Montemurro, Sands, et al., 2016) despite significantly increasing genioglossus activity during sleep (Taranto‐Montemurro, Edwards, et al., 2016; Taranto‐Montemurro, Messineo, & Wellman, 2019). In comparison, trials using serotonergic agents to treat OSA have shown greater reductions in the apnoea‐hypopnoea index (AHI): up to 40% in some cases (Taranto‐Montemurro, Messineo, & Wellman, 2019) and may still have a significant beneficial effect toward increasing genioglossus muscle tone (Kubin, 2016). Notably, animal data have shown that pharmacologically augmenting both of these monoamines has an additive effect toward increasing pharyngeal muscle tone (Chan et al., 2006). However attempts to replicate these data in human studies using the serotonin‐norepinephrine reuptake inhibitor, venlafaxine, has had limited success in reducing OSA severity (Schmickl et al., 2020).

The limited results of these monoaminergic‐enhancing treatment approaches could be partly due to inadequately accounting for the cholinergic inhibition (via muscarinic receptors) of the pharyngeal motor nuclei that leads to genioglossus atonia during REM sleep (Grace et al., 2013). Recent trials have demonstrated that the combination of broad‐spectrum noradrenergic reuptake inhibitors (NRI) with an antimuscarinic can substantially reduce airway collapsibility and improve the AHI in OSA patients (Lim et al., 2021; Perger et al., 2022; Taranto‐Montemurro, Messineo, Sands, et al., 2019). It is unknown how effective other dual reuptake inhibitors will be when combined with an antimuscarinic.

Like the noradrenergic agent's atomoxetine and reboxetine, it is possible that serotonergic agents also require a paired antimuscarinic to be more efficacious. To further explore the mechanisms behind successful monoaminergic/antimuscarinic combination therapy, the current study aimed to investigate the effect of oxybutynin on OSA when administered with either milnacipran, a norepinephrine‐serotonin reuptake inhibitor (NSRI) or duloxetine, a serotonin‐norepinephrine reuptake inhibitor (SNRI). Milnacipran and duloxetine were selected as they have comparable pharmacokinetics, however, milnacipran has a stronger noradrenergic effect than duloxetine which preferentially and more potently targets serotonin (Kasper & Pail, 2010). Lastly, comparing these two agents will also help further explore the utility of increasing serotonin in managing OSA. If it is true that norepinephrine contributes more to increased genioglossus muscle tone, then it is expected that milnacipran+oxybutynin will produce greater improvements in airway collapsibility and reductions in OSA severity compared to duloxetine+oxybutynin.

## 2 METHODS

### 2.1 Ethics statement

The study was performed in accordance with the ethical principles as outlined in the Declaration of Helsinki for medical research involving humans. Written informed consent was obtained prior to study commencement. The trial was approved by the Monash University Human Research Committee (#16795) and was prospectively registered with the Australian New Zealand Clinical Trials Registry (ACTRN12618001499279).

### 2.2 Participants

Overall, eleven individuals with OSA were recruited via public advertising and telephone screening between January and June, 2019. One participant withdrew citing personal reasons. Enrolled candidates were required to have an AHI >20 events/h, as indicated by a previous polysomnography (PSG) study. Those receiving any current OSA treatment (e.g. CPAP) were asked to abstain for at least 5 nights prior to each PSG study. Exclusion criteria included any comorbid medical condition other than well‐controlled hypertension, diabetes and hyperlipidemia; BMI >35 kg/m^2^; Epworth Sleepiness Scale (ESS) score > 16; a central apnoea index >5 events/h; inability to sleep supine; pregnancy or breastfeeding; known allergies or taking medications contraindicated to use with the investigational products (oxybutynin, duloxetine, and milnacipran); or taking medications known to influence sleep, respiration, or muscle physiology. The trial concluded when 10 enrolled participants completed all study nights.

### 2.3 Protocol

A randomized, double‐blind, 4‐way crossover, placebo‐controlled trial was performed to assess the efficacy of dual reuptake inhibitors with antimuscarinic combination therapy on the primary outcome of OSA severity as measured by the AHI. Secondary outcomes were cardiovascular functioning (blood pressure/heart rate), both subjective and objective sleep quality, and other indices of OSA severity (such as overnight oxygen desaturation and arousals from sleep). All outcomes were assessed over four overnight in‐lab PSG studies were performed approximately 1‐week apart where participants were randomized to take each of the following combinations: (1) milnacipran (50 mg) + oxybutynin (5 mg) [Mil‐Oxy], (2) duloxetine (60 mg) + oxybutynin (5 mg) [Dul‐Oxy], (3) oxybutynin (5 mg), (4) placebo. These doses were chosen based on commonly prescribed doses for other approved conditions. Study medications were prepared by an independent compounding pharmacy and packaged as identical capsules. Randomization of treatment conditions was performed using an online sequence generator by an investigator not involved in participant enrolment, data collection or outcome assessment.

For each visit, participants arrived at the Monash University Sleep Laboratory at 19:00 h. Anthropometric measurements were taken (height, weight and neck/waist circumference were recorded during the inaugural lab visit; weight was recorded upon arrival at subsequent visits) before staff assessed any adverse events that may have occurred during washout. Participants were then instrumented with PSG and respiratory equipment, as detailed below. Blood pressure was measured (as triplicate) 10 min before bedtime in both the seated and supine position preceding 5 min of quiet restful breathing.

The study medications were given prior to lights out as determined by participants' habitual bedtime and kept consistent between conditions. Given that the pharmacokinetic profiles of each active drug are not homogeneous, to ensure peak drug concentration occurred during the middle of each sleep period, duloxetine or milnacipran were administered 2 h before habitual bedtime and oxybutynin was administered 30 min prior.

Participants were then given an 8‐h opportunity to sleep in which at least 50% of the recording was in the supine position. Blood pressure was measured as described 10 min post‐awakening and subjective sleep quality was then assessed using the Stanford Sleepiness Scale and a 10‐point visual analogue scale (1 indicating poor sleep quality, 10 indicating optimal sleep quality). Any side effects were recorded by staff before discharge from the laboratory.

### 2.4 Equipment and measurements

A standard clinical montage for OSA evaluation was used comprising six channel electroencephalogram (EEG), bilateral electrooculogram, submental and anterior tibialis electromyogram, electrocardiogram, thoracic and abdominal respiratory inductance plethysmography bands, finger pulse oximetry, tracheal microphone and body position. Body position was confirmed using an infrared camera and corrected during analysis if required. Airflow and pressure was measured using a sealed oronasal mask (Resmed Airfit F10) connected to a pneumotachometer (model 3700A; Hans‐Rudolph) and differential pressure transducer (Hans‐Rudolph) referenced to atmosphere. End‐tidal CO_2_ and O_2_ were continuously sampled from one nostril (Vacumed). All signals were recorded at 256 Hz and displayed using the Profusion PSG (v4 Compumedics) software.

### 2.5 Data analysis

Sleep stages, arousals and respiratory events were scored according to standard criteria by an experienced sleep scientist blinded to treatment conditions. Hypopnoeas were scored using the AASM 2012 recommended criteria (i.e. as a reduction in the airflow signal ≥30% for at least 10 seconds with an accompanying oxygen desaturation ≥3% or an EEG arousal, Berry et al., 2012). Once the PSG data were scored the hypoxic burden was also extracted, which measures the impact of arterial hypoxaemia by calculating the cumulative ‘area under the curve’ of oxygen desaturations resulting from individual apnoeas/hypopnoeas and then divided by sleep duration ([%min]/h, Azarbarzin et al., 2019). A log base 10 was used to transform the data as previously reported (Azarbarzin et al., 2019).

#### 2.5.1 Endotypes of OSA

Measures of the OSA endotypes (airway collapsibility, muscle responsiveness, loop gain, and respiratory arousal threshold) were derived from the acquired PSG traces using the validated techniques described previously (Sands, Edwards, Terrill, Taranto‐Montemurro, et al., 2018; Sands, Terrill, et al., 2018; Terrill et al., 2015). Briefly, the PSG was divided into 7‐min windows in order to estimate breath‐by‐breath measurements of ventilatory drive (*V*_drive_) by fitting a standard ventilatory control model (that consist of a gain, time constant and delay) to the observed ventilation. The gain term represents the loop gain (here we report the response to a 1‐cycle/min disturbance), where a higher loop gain reflects a more unstable ventilatory control system. The model also fits an additional parameter in order to capture the ‘wakefulness’ ventilatory drive during scored EEG arousals (i.e., ventilatory response to arousal; VRA). Once ventilatory drive is known, all other endotypes are extracted. The arousal threshold is measured as the median value of *V*_drive_ immediately preceding that of a scored respiratory related arousal (Sands, Terrill, et al., 2018). Measurements of airway collapsibility (*V*_passive_, *V*_min_ and *V*_active_) are expressed as a percentage of median eupneic ventilation (*V*_eupnea_; when ventilation and *V*_drive_ are matched). *V*_passive_ is the level of ventilation at eupneic ventilatory drive and reflects the passive airway, while *V*_active_ is the ventilation achieved just prior to the termination of a respiratory event when *V*_drive_ is at its peak and the airway is maximally stimulated. The *V*_min_ provides an alternative measure of collapsibility and is based on the ventilation observed at the minimal level of *V*_drive_ (calculated from the lowest decile instead of median *V*_eupnea_, Vena et al., 2022). A lower value denotes a more collapsible airway. Data for both *V*_passive_ and arousal threshold were square root transformed as previously reported (O'Driscoll et al., 2019; Sands, Edwards, Terrill, Butler, et al., 2018).

### 2.6 Statistical analyses

Statistical analyses were performed using GraphPad Prism 8.4.3 (GraphPad software, San Diego, California USA). This was an exploratory study in which these drugs were tested for the first time in OSA patients, we hypothesized based on trial data from a previous study with similar drugs (Taranto‐Montemurro, Messineo, Sands, et al., 2019) a sample size of 10 patients would detect a difference of 18 (±18) events/h (assuming 1‐*β* = 0.8, *α* = 0.05), allowing for 20% attrition rate. Between‐condition comparisons were performed using linear mixed effect modeling or Friedman tests depending on whether data were normally distributed (as determined using Shapiro–Wilk tests). Due to the small sample size and exploratory nature of this pilot study, post‐hoc tests were performed using Fisher's Least Significant Difference (paired to placebo) or an uncorrected Dunn's test for parametric and non‐parametric data respectively. Effect sizes were determined using Cohen's D calculations. We also explored the relationships between changes in AHI and key endotype variables for both the Dul‐Oxy and Mil‐Oxy conditions using simple linear regression. All main analyses were performed using data recorded during NREM sleep in the supine position. Other sleep state/body position combinations were also analyzed as a sensitivity analysis (see online supplement). Data are reported as means (±SD) or median [Q1–Q3] as appropriate. Statistical significance was inferred at *p* ≤ 0.05.

## 3 RESULTS

### 3.1 Participant details

**FIGURE 1:**
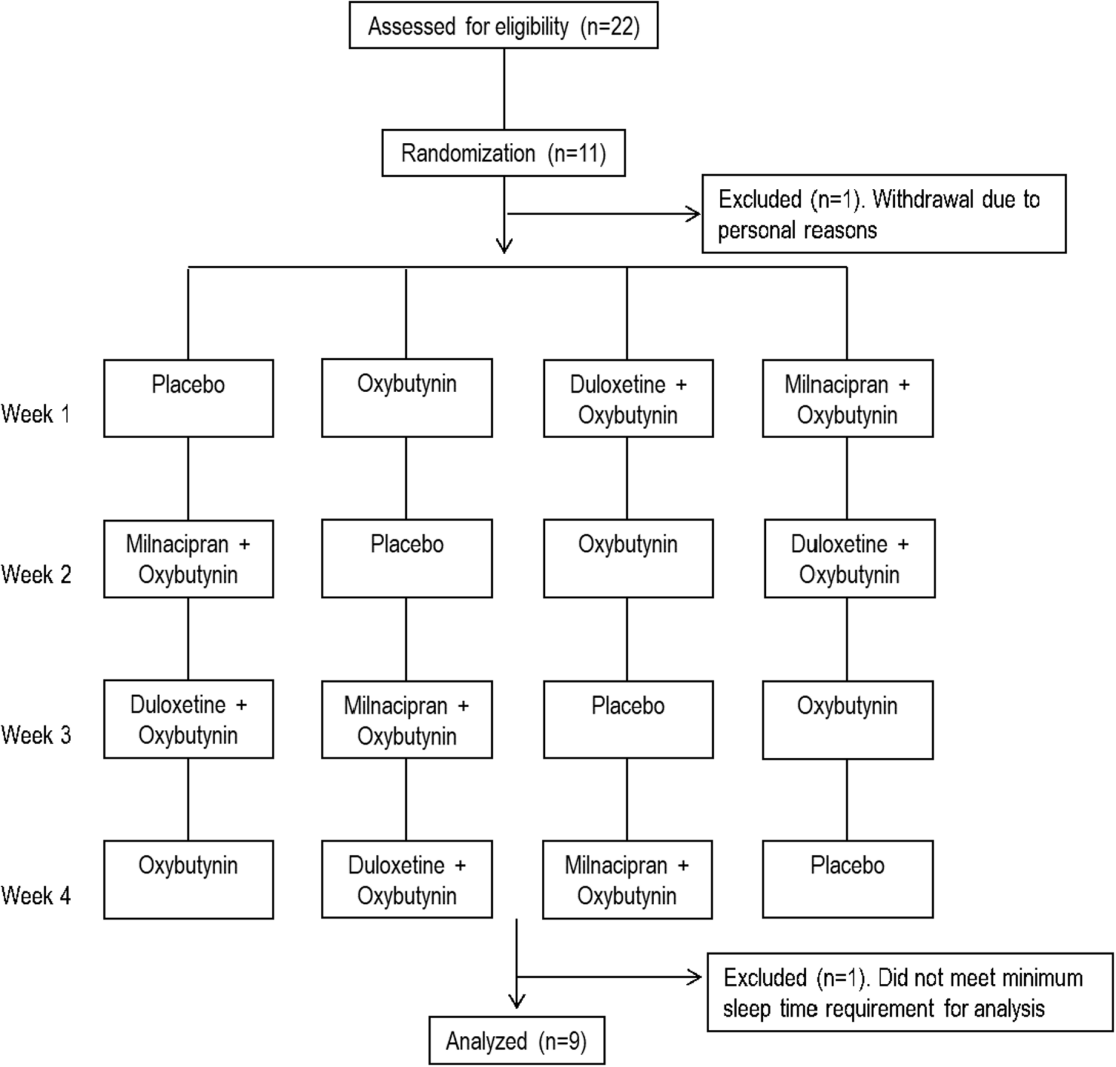
Consort diagram of the clinical trial.

Eleven participants were initially enrolled in the study; however, one withdrew for personal reasons (see Figure 1). Of the ten patients that completed the study protocol, one participant routinely slept less than 2 h (mean TST = 104 mins; range: 72–175 mins) and was therefore excluded from the final analysis as not enough PSG data were obtained to quantify sleep and respiratory variables accurately. Data were analyzed in the remaining nine participants whose characteristics are outlined in Table 1. On average the participants were middle aged, overweight with severe OSA and moderate airway crowding (Mallampati >3). Three of the participants were currently treated with CPAP with an average self‐reported compliance of 7.3 h across 6.5 nights/wk. Known side effects of the investigational drugs were recorded by five of the original ten participants: two of whom reported mild‐to‐moderate nausea in both the Dul‐Oxy and Mil‐Oxy conditions, and three others separately experienced a dry mouth after taking oxybutynin (*n* = 1), Dul‐Oxy (*n* = 1) and Mil‐Oxy (*n* = 1).

**TABLE 1:** Participant demographics

Age (years)	55 ± 6.2
Sex (Female: male)	4:5
BMI (kg/m^2^)	30 ± 2.8
Neck circumference (cm)	40 ± 3.4
AHI (events/hr)	31 ± 14
ESS	9.3 ± 4.2
Mallampati classification	3.3 ± 1.1
Race, *n* (%)	
Caucasian	5 (55.5)
Asian	3 (33.3)
Other	2 (22.2)
Comorbidities, *n* (%)	
Hypertension	3 (33.3)
Diabetes	1 (11.1)
Depression/Anxiety	3 (33.3)
Hypercholesterolemia	1 (11.1)
Arthritis	1 (11.1)
Gout	1 (11.1)
GORD	2 (22.2)
Medications, *n* (%)	
ACE‐I/ARB	2 (22.2)
β‐Blockers	1 (11.1)
Antidiabetics	1 (11.1)
Antilipemic	1 (11.1)
Xanthine oxidase inhibitor	1 (11.1)
Protonic pump inhibitor	2 (22.2)

*Note*: Data presented as the mean ± SD; *n* = 9.

Abbreviations: ACE‐I, angiotensin‐converting enzyme inhibitor; AHI, apnoea hypopnoea index; ARB, angiotensin receptor blocker; BMI, body mass index; ESS, Epworth Sleepiness Scale; GORD, Gastro‐esophageal reflux disease.

### 3.2 Effect of drug combinations on OSA severity and sleep characteristics

**FIGURE 2:**
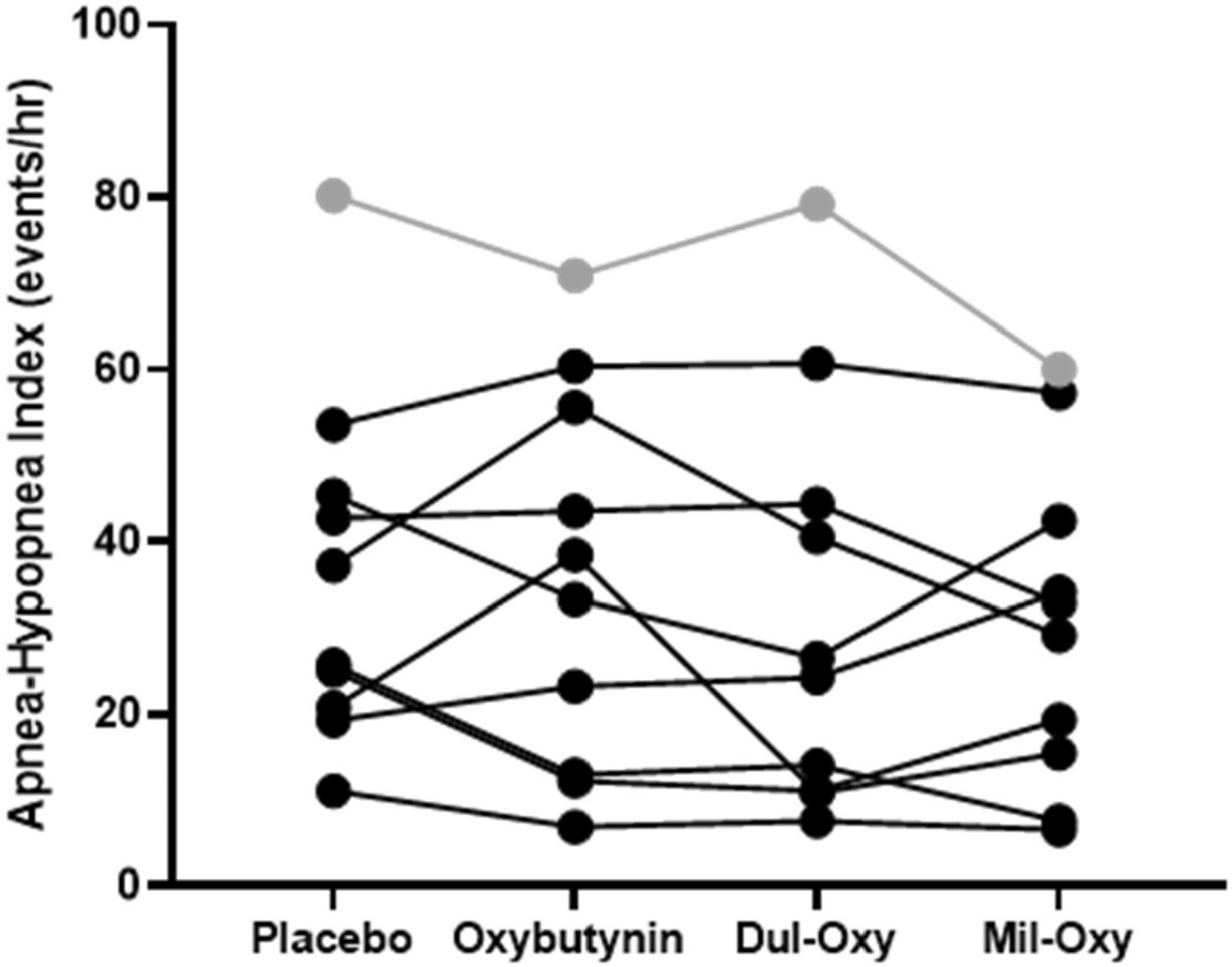
The total apnoea‐hypopnoea index between all conditions (oxybutynin, duloxetine+oxybutynin [Dul‐Oxy] and milnacipran+oxybutynin [Mil‐Oxy]) compared with placebo as intention‐to‐treat (*n* = 10) expressed as individual data (circles). Gray circles shows the participant excluded due to habitual short sleep.

Neither of the drug combinations (Dul‐Oxy [*p* = 0.18; *d* = 0.25] or Mil‐Oxy [*p* = 0.23; *d* = 0.26]) significantly altered the total AHI across the patients in this study, regardless of whether it was analyzed as intention to treat (*n* = 10) or per protocol (*n* = 9, see Figure 2). There was also no change in the NREM supine AHI (see Table 3) or any other AHI combinations of sleep state/body position (see online supplement). However, Dul‐Oxy did improve the fraction of hypopnoeas to apnoeas (*F*_Hypopnoea_) compared to placebo (*p* = 0.02; *d* = 0.54) suggesting an improvement in the severity of airway obstruction. Neither drug combination showed significant improvement in any measurements related to nocturnal arterial blood oxygen (the average, nadir, or index of SpO_2_ desaturations) or hypoxic burden. Although post‐hoc exploratory analysis did reveal an improved hypoxic burden in the Dul‐Oxy condition when compared to placebo (*p* = 0.02; *d* = 0.56). Mil‐Oxy increased the morning heart rate (*p* = 0.05; *d* = 0.58) and diastolic pressure (*p* = 0.003; *d* = 0.55) and there was a group level trend toward increased diastolic blood pressure at lights out in both the Dul‐Oxy (*p* = 0.05; *d* = 0.62) and Mil‐Oxy (*p* = 0.009; *d* = 0.77) conditions compared to placebo (see Table 2).

**TABLE 2:** Effects of treatment drugs on sleep and cardiovascular parameters compared with placebo

	Placebo	Oxybutynin	Dul‐Oxy	Mil‐Oxy	*p* value
Total sleep time (mins)	388 ± 60	388 ± 44	387 ± 36	384 ± 44	0.93
NREM supine sleep (%TST)	49 ± 26	52 ± 24	49 ± 26	49 ± 24	0.96
Sleep efficiency (%)	82 ± 13	81 ± 8.8	83 ± 7.0	79 ± 9.0	0.65
Sleep latency (mins)	15 ± 15	19 ± 16	11 ± 10	22 ± 22	0.29
WASO (mins)	53 [34―89]	68 [38―92]	53 [44―88]	92 [48―104]	0.90
NREM (%TST)	90 ± 7.4	90 ± 9.3	96 ± 4.2***	90 ± 8.6	0.004
N1 (%TST)	18 ± 6.9	22 ± 6.8	29 ± 8.8***	24 ± 10	0.01
N2 (%TST)	52 ± 8.2	55 ± 3.7	57 ± 7.2	52 ± 7.7	0.17
N3 (%TST)	19 ± 12	12 ± 5.8	10 ± 4.8	14 ± 6.5	0.09
REM (%TST)	10 ± 7.4	10 ± 9.3	3.8 ± 4.2***	10 ± 8.6	0.004
Arousal Index (arousals/h)					
Total	31 ± 11	30 ± 9.4	29 ± 7.8	28 ± 11	0.60
Respiratory	16 ± 6.8	15 ± 8.6	12 ± 7.7	15 ± 11	0.30
Morning alertness	3 [2―3]	2.5 [1.8―3.0]	2.0 [2.0―4.0]	2.5 [2.0―3.3]	0.13
Subjective sleep quality	5.6 ± 18	5.6 ± 2.1	5.7 ± 1.1	5.2 ± 2.1	0.87
Evening heart rate (beats/min)	62 ± 8.9	67 ± 12	62 ± 11	66 ± 12	0.50
Morning heart rate (beats/min)	65 ± 8.2	63 ± 11	66 ± 15	72 ± 15*	0.01
Blood pressure (supine)					
Evening systolic (mmHg)	124 ± 6.2	125 ± 6.8	128 ± 5.7	135 ± 18	0.13
Morning systolic (mmHg)	130 ± 13	129 ± 9.6	132 ± 14	138 ± 10	0.20
Evening diastolic (mmHg)	73 ± 6.7	74 ± 7.5	77 ± 6.2*	80 ± 11**	0.07
Morning diastolic (mmHg)	78 ± 9.9	79 ± 9.5	79 ± 8.8	84 ± 12**	0.03

*Note*: Sleep efficiency measures time asleep/total sleep period; Sleep latency measures time taken to sleep onset; WASO, wake after sleep onset; NREM, nonrapid eye movement; REM, rapid eye movement; N1, Stage 1 Sleep; N2, Stage 2 Sleep; N3, Stage 3 Sleep; Morning alertness was measured with the Stanford Sleepiness Scale, a score above 3 was considered sleepy; Subjective sleep quality was measured using a visual analogue scale, a higher score indicates higher sleep satisfaction; Dul‐Oxy, duloxetine+oxybutynin; Mil‐Oxy, milnacipran+oxybutynin. Data presented as means ± SD or median [interquartile range]. *n* = 9. Asterisks defined below indicate significant differences relative to placebo (post‐hoc comparison).

* *p* ≤ 0.05

** *p* ≤ 0.01

*** *p* ≤ 0.001.

**TABLE 3:** Effects of treatment drugs on markers of sleep apnea severity and endotypes

	Placebo	Oxybutynin	Dul‐Oxy	Mil‐Oxy	*p* value
AHI (events/h)					
Total	31 ± 14	32 ± 19	27 ± 18	27 ± 17	0.36
REM	48 ± 23	44 ± 22	46 ± 30	34 ± 27	0.28
NREM	29 ± 14	30 ± 20	26 ± 18	26 ± 17	0.59
NREM supine	50 [29―53]	53 [27―63]	34 [16―51]	43 [14―62]	0.51
*F*_Hypopnoea_					
Total	67 ± 17	72 ± 17	81 ± 16**	68 ± 21	0.009
NREM supine	71 ± 22	78 ± 17	82 ± 21*	71 ± 24	0.18
Event duration – total (s)	24 [17―27]	22 [16―24]	20 [16―24]	19 [17―23]*	0.04
Nadir SpO_2_ desaturation (%)	83 [78―89]	87 [82―90]	87 [84―90]	86 [83―91]	0.14
Average SpO_2_ desaturation (%)	5 [4―6]	5 [3.5―6]	4 [3.5―5]	5 [3.5―6.5]	0.15
Total ODI 3%	25 ± 12	24 ± 17	22 ± 18	22 ± 13	0.28
Total ODI 4%	21 [7.1―24]	10 [3.9―32]	6.3 [3.5―28]	15 [8.1―18]	0.35
Hypoxic burden ([%min]/h) Log10	1.7 ± 0.35	1.7 ± 0.45	1.5 ± 0.36*	1.6 ± 0.41	0.11
Loop gain	0.68 [0.58―0.75]	0.64 [0.57―0.92]	0.64 [0.57―0.75]	0.65 [0.60―0.73]	0.44
VRA (%V_eupnea_)	30 [21―59]	32 [21―36]	23 [13―30]*	24 [17―48]*	0.04
ArTH (%V_eupnea_)	156 ± 20	157 ± 22	145 ± 30	150 ± 21	0.31
V_passive_ (%V_eupnea_)	69 [59―79]	64 [52―77]	72 [63―95]	72 [54―85]	0.11
V_active_ (%V_eupnea_)	113 ± 19	102 ± 34	104 ± 18	97 ± 31	0.30
V_min_ (%V_eupnea_)	58 ± 15	57 ± 16	65 ± 17	60 ± 14	0.34

*Note*: REM AHI calculated only if REM sleep was achieved in all conditions (*n* = 7). Data presented as means ± SD or median [interquartile range]. *n* = 9.

Abbreviations: AHI, apnoea‐hypopnoea index; ArTH, arousal threshold; Dul‐Oxy, duloxetine+oxybutynin; F_Hypopnoea_, the amount of all respiratory events that were hypopnoeas as a fraction of total respiratory events; Log10, log‐transformed, base 10; Mil‐Oxy, milnacipran+oxybutynin; NREM, nonrapid eye movement; ODI, oxygen desaturation index; SpO_2_, peripheral capillary oxygen saturation; V_active_, ventilation when upper airway dilator muscles are maximally activated; Veupnea, eupneic ventilation; V_min_, ventilation at lowest ventilatory drive during the respiratory events; V_passive_, ventilation when upper airway dilator muscles are hypotonic/passive; VRA, ventilatory response to arousal. Asterisks defined below indicate significant differences relative to placebo (post‐hoc comparison).

* *p* ≤ 0.05

** *p* ≤ 0.01;

***p ≤ 0.001.

**FIGURE 3:**
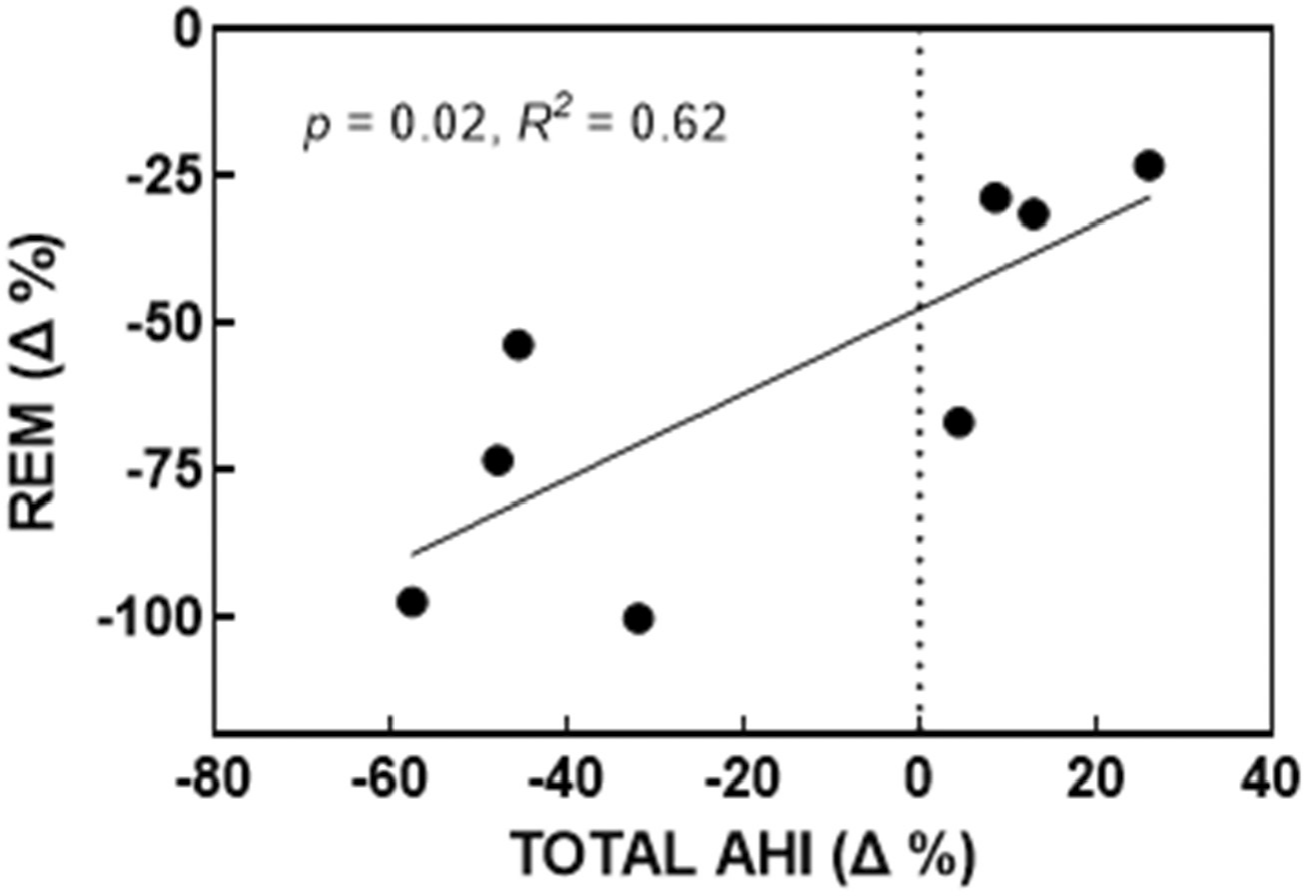
Effect of duloxetine+oxybutynin on time spent in REM sleep (% change relative to placebo) by the total AHI (% change from placebo). People who experienced a greater reduction in REM sleep also experienced a greater reduction in their total AHI. Findings indicate that the reduction in AHI is more driven by a reduction in REM sleep for these individuals. One participant did not achieve any REM sleep over all four conditions and thus was excluded from the analyses. *N* = 8.

There were no differences between drug conditions in regard to sleep quantity or quality (total sleep time, sleep latency, sleep efficiency or wake after sleep onset; see Table 2). There was also no difference in the amount of time spent in the supine position during NREM sleep between all conditions (49–52%; *p* = 0.96). Sleep architecture was different between placebo and Dul‐Oxy which promoted more NREM sleep (*p* = 0.009; *d* = 0.99); specifically, it increased time spent in NREM1 (*p* = 0.006; *d* = 1.39) at the expense of reduced time in REM (*p* = 0.009; *d* = 1.03). This reduction in REM sleep was positively associated with a reduction in the total AHI (*R* ^2^ = 0.62; *p* = 0.02; see Figure 3). There were no significant differences between drug conditions in terms of subjective sleep quality or next day alertness.

### 3.3 Effect of drug combinations on the OSA endotypes

**FIGURE 4:**
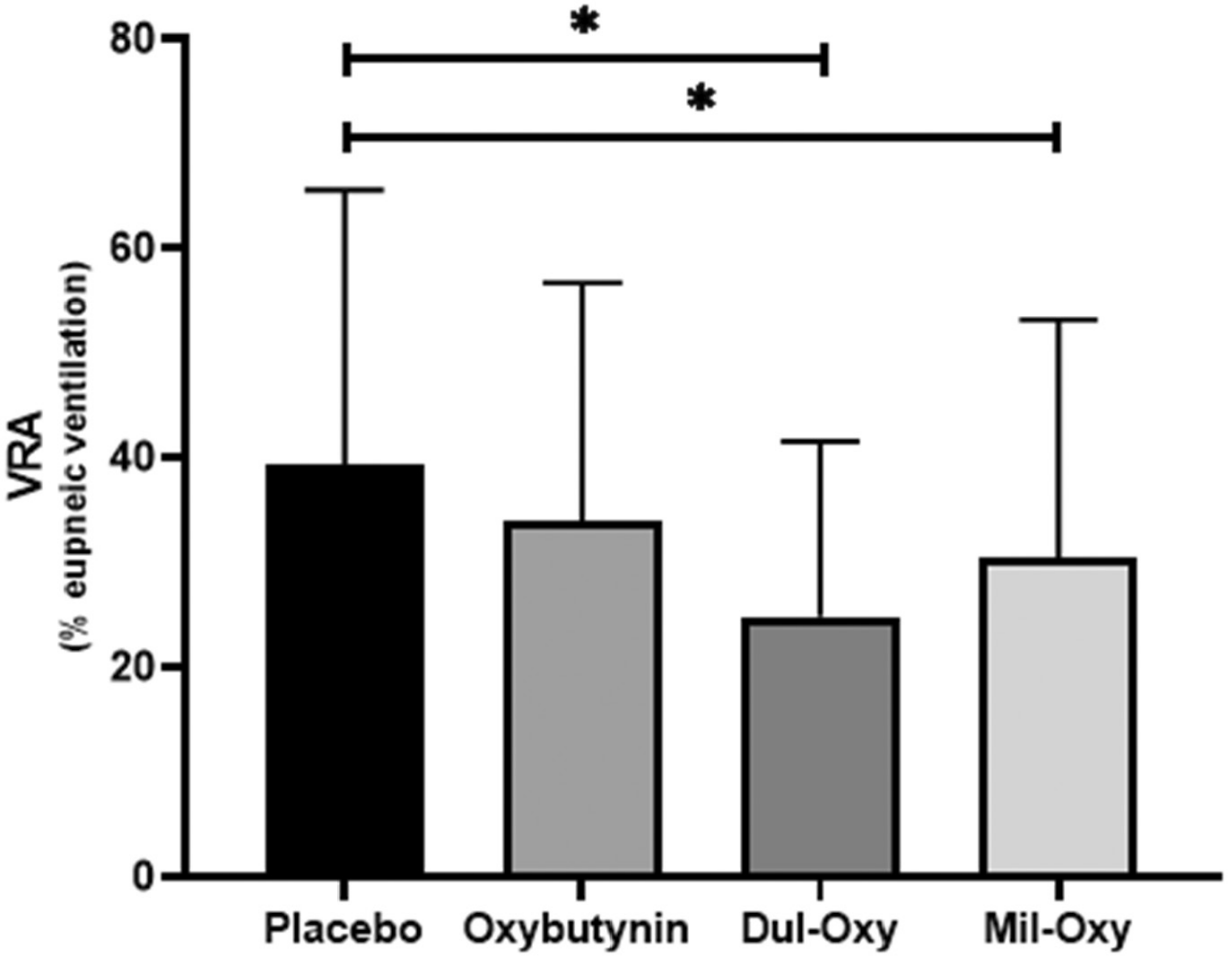
Effect of drug conditions between all conditions (oxybutynin, duloxetine+oxybutynin [Dul‐Oxy] and milnacipran+oxybutynin [Mil‐Oxy]) on the ventilatory response to arousal (VRA) compared with placebo. Bar graphs represent mean with confidence intervals. Pairwise comparisons were performed using uncorrected Fisher's least significant difference test. *p < 0.05. *N* = 9.

Similar to the impact on OSA severity, the drug combinations did not significantly affect any of the OSA endotypes (loop gain, arousal threshold, *V*_passive_, *V*_active_ or *V*_min_). However, there was a significant group level difference in the VRA showing it was reduced in both the Dul‐Oxy (*p* = 0.03; *d* = 0.63) and Mil‐Oxy (*p* = 0.02; *d* = 0.37) conditions compared to placebo (see Figure 4). In order to assess whether the change in endotypes impacted the AHI, linear regressions were conducted. For the Dul‐Oxy condition, a greater reduction in the arousal threshold was associated with a greater reduction in the total AHI from placebo (*R* ^2^ = 0.56; *p* = 0.02). Improvements in *V*_passive_ (*R* ^2^ *=* 0.62; *p* = 0.01) and *V*_active_ (*R* ^2^ = 0.62; *p* = 0.01) during NREM supine sleep were associated with a reduction in the NREM supine AHI for the Mil‐Oxy condition only. There was also a trend for greater improvements of *V*_min_ (*R* ^2^ = 0.40; *p* = 0.07) in the Dul‐Oxy condition during NREM supine sleep to be associated with greater reductions in the NREM supine AHI. A higher *V*_min_ (i.e. less collapsible airway) during placebo was also predicative of a lower total AHI during treatment for both Dul‐Oxy (*R* ^2^ = 0.67; *p* = 0.007) and Mil‐Oxy (*R* ^2^ = 0.55; *p* = 0.02). No other associations were demonstrated between the endotypes and AHI.

## 4 DISCUSSION

This is the first study to combine dual norepinephrine/serotonin reuptake inhibitors (duloxetine and milnacipran) with an antimuscarinic (oxybutynin) to test the effect of these combinations on OSA severity and the underlying OSA endotypes. The main findings were that a single dose of either Dul‐Oxy (60 mg + 5 mg) or Mil‐Oxy (50 mg + 5 mg) administered before bedtime did not significantly alter the AHI compared to placebo. However, Dul‐Oxy did significantly increase the *F*_Hypopnoea_ (a higher number of respiratory events shifted from complete to only partial obstructions) suggesting an improvement in airway collapsibility. Nonetheless, overall neither of the drug combinations improved the OSA endotypes. While both combinations did significantly lower the VRA, this reduction did not appear to have any impact on OSA severity for the participants in this study.

### 4.1 Impact of the drug combinations on OSA severity

Unlike previous trials combining norepinephrine‐specific agents with an antimuscarinic; neither of the dual reuptake inhibitors combined with oxybutynin in the current study were able to significantly impact the AHI, regardless of whether the combination preferentially increased serotonin (Dul‐Oxy) or norepinephrine (Mil‐Oxy). Likewise, there were also no improvements in markers of nocturnal blood oxygen (SpO_2_ nadir, ODI, hypoxic burden etc) at the group level, although exploratory pairwise comparisons suggested that Dul‐Oxy may have improved the hypoxic burden compared to placebo. Similarly, we found that there was a large effect size reduction in the ODI4% (compared to placebo [−14.7 events/h; *d* = 0.91]). This may indicate that while Dul‐Oxy does not substantially reduce the incidence of respiratory events; it may decrease the depth of events (i.e., improved *F*_Hypopnoea_) and the degree of blood oxygen desaturation that occurs (i.e., reduced ODI4% and hypoxic burden).

The overall limited effect of these drug combinations on improving OSA severity are consistent with previous trials seeking to treat OSA by pharmacologically increasing either norepinephrine and/or serotonin (Taranto‐Montemurro, Messineo & Wellman, 2019). The more successful results of the atomoxetine+oxybutynin trial (Taranto‐Montemurro, Messineo, Sands, et al., 2019; Taranto‐Montemurro et al., 2020) gave reason to investigate these previously trialed monoaminergic agents when co‐administered with an antimuscarinic. The results of this pilot study suggest instead that a potent noradrenergic‐specific agent with broad‐spectrum acting properties (such as atomoxetine or reboxetine) is preferred for the antimuscarinic + norepinephrine reuptake inhibitor combination to provide a significant improvement in OSA severity.

### 4.2 Impact of the drug combinations on sleep

Compared to placebo, there were no differences in total sleep time or sleep efficiency between the drug conditions. As expected, the Dul‐Oxy condition decreased REM by 62%. A similar magnitude reduction has been observed when atomoxetine was administered with solifenacin succinate or biperiden (Aishah et al., 2021) as well as when reboxetine was administered with hyoscine butylbromide (Lim et al., 2021). Oxybutynin has also been shown to decrease REM sleep by approximately 20% when taken alone and with atomoxetine (Taranto‐Montemurro et al., 2020). Given that noradrenergic agents and antimuscarinics reduce REM sleep (when taken either alone or in combination), it is unexpected that this reduction was not observed in the Mil‐Oxy condition. Furthermore, in contrast to previous findings (Lim et al., 2021), the reduction in REM sleep caused by Dul‐Oxy was strongly associated with a reduction in the total AHI. OSA can be more severe during REM sleep and pharmacologically induced REM sleep suppression has been offered as a potential OSA therapy, although with potentially deleterious consequences to cognition (Lim et al., 2021).

### 4.3 Impact of the drug combinations on the OSA endotypes

Contrary to our hypothesis, there was no significant effect of the drug combinations on the measures of collapsibility. Moreover, despite oxybutynin improving *V*_passive_ by 18% in a previous trial (Taranto‐Montemurro et al., 2020), oxybutynin alone had no impact on airway collapsibility in the current trial. Previous trials administering a single‐night dose of a noradrenergic/antimuscarinic drug combination demonstrated significant improvements in airway collapsibility and a ~10%–15% reduction in loop gain which was largely attributed to the effect of increasing norepinephrine (Lim et al., 2021; Taranto‐Montemurro et al., 2020). Conversely, we did not find a similar effect of Mil‐Oxy on either collapsibility or loop gain. Interestingly, the combination of reboxetine and oxybutynin also had no effect on loop gain and collapsibility following 1 week of administration suggesting that this may be an artifact of acute dosing (Perger et al., 2022).

From a pharmacodynamic perspective, the NSRI, milnacipran, is similar to atomoxetine and reboxetine in that they are all potent broad‐spectrum acting agents (with no affinity for specific receptors) that increase norepinephrine by preventing its reuptake (Kasper & Pail, 2010). Human studies have shown that a single dose (50 mg) of milnacipran is effective at increasing extracellular levels of norepinephrine, although doubling the dose to 100 mg is required to achieve the efficacy similar to tricyclics (Shelton, 2019). Further, patients in the Mil‐Oxy condition had significantly increased morning blood pressure and heart rate, a typical noradrenergic effect which gives confidence that milnacipran did increase norepinephrine levels. One major difference between atomoxetine, reboxetine, and milnacipran is that milnacipran also increases extracellular serotonin by blocking its reuptake. It is known that systemic administration of serotonin can have contradicting effects on ventilatory drive and can actually lower airway muscle activation (Millhorn et al., 1983; Veasey, 2003) which may explain why the Mil‐Oxy combination lacked the efficacy in this study to reduce the AHI, and also non‐significantly decreased neuromuscular compensation by 14% from placebo.

Based on previous data showing norepinephrine to be more instrumental than serotonin toward improving airway collapsibility in humans (Fenik et al., 2005), we expected the NSRI, milnacipran, to outperform the SNRI, duloxetine. However, we found that only Dul‐Oxy showed signs of potentially improving airway collapsibility as demonstrated by a significant increase in %_Hypopnoea_ (and the trend for Dul‐Oxy to improve the *V*_min_) compared to placebo. Moreover, Dul‐Oxy had a stronger effect on OSA severity than Mil‐Oxy, demonstrated by a greater (albeit non‐significant) decrease in the NREM‐supine AHI; 32% compared to 14%, respectively. These two drugs are pharmacokinetically similar in terms of half‐life (8 h), bio‐availability (85%) and maximum plasma concentration (2 h), and both drugs penetrate the blood brain barrier (Shelton, 2019); however, they are pharmacodynamically distinct in that duloxetine has a 10 times greater binding affinity for serotonin and is 3.5 times more potent in blocking its reuptake as it is in blocking norepinephrine reuptake (Kasper & Pail, 2010; Shelton, 2019). Furthermore, the effects of duloxetine's ability to sufficiently increase norepinephrine are questionable, especially at low doses (<80 mg) which were shown to have no noradrenergic effect in healthy humans (Chalon et al., 2003; Turcotte et al., 2001)^.^ While not conclusive, our results suggest that modifying both neuromodulators may have contravening effects that can disadvantage the airway more than if just one was targeted. This is worthy of more consideration if noradrenergic+antimuscarinic combination therapies are to be administered to OSA patients taking a selective serotonin reuptake inhibitor for conditions such as co‐morbid depression.

There was a significant median decrease in the VRA compared to placebo by 23% for Dul‐Oxy, and 20% for Mil‐Oxy. Blunting the VRA may be beneficial toward alleviating OSA in some patients; however, lowering the VRA did not lead to any improvement in OSA severity for either drug combination. Schmickl and colleagues (Schmickl et al., 2020) recently showed the SNRI, venlafaxine, lowered the VRA which was the first trial to report a monoaminergic drug having this effect. In contrast to the current trial, venlafaxine yielded a much larger reduction in the VRA (39%) which was correlated with the reduction in AHI. It is therefore possible that the more modest reductions in VRA elicited by Dul‐Oxy and Mil‐Oxy did not reach a threshold necessary for a lower VRA to effectively contribute to a reduction in OSA severity. Taken together, the results of these trials suggest that augmenting serotonin may be a key mechanism to pharmacologically reducing the magnitude of the VRA.

We had hypothesized the arousal threshold would be lower compared to placebo due to the increased alerting properties of augmenting serotonin and norepinephrine, which was reported in the original atomoxetine+oxybutynin trial (Taranto‐Montemurro, Messineo, Sands, et al., 2019; Taranto‐Montemurro et al., 2020). However, the arousal threshold was not changed in either drug condition. This is more consistent with other trials (Aishah et al., 2021; Lim et al., 2021) and is likely due to the addition of oxybutynin. It has been speculated that oxybutynin may increase the arousal threshold due to a mild sedating effect (Taranto‐Montemurro et al., 2020). Indeed, oxybutynin was found to limit the stimulating properties of atomoxetine and increase the arousal threshold when taken in combination, rather than alone (Taranto‐Montemurro, Messineo, Sands, et al., 2019).

### 4.4 Methodological considerations

The current trial employed a randomized, placebo‐controlled, double blinded study design that included a wider spread of AHI severity and more women that have been previously reported for similar drug trials of this nature. Although we were well‐powered to detect a change in the primary outcomes of OSA severity, the exploratory nature of this pilot study combined with its smaller sample size limits the conclusions we can draw about the secondary findings addressing potential underlying mechanisms. Moreover, without the use of blood plasma testing, we are not able to confidently detect whether the drugs were biologically utilized to their full potential and if they had a uniform effect on all participants at standard dosing. The physiological effects of monoaminergic agents are also well known to change over time and so chronic dosing studies within a larger and more targeted sample would be needed to draw firmer conclusions.

## 5 CONCLUSION

In conclusion, neither dual reuptake inhibiter (milnacipran or duloxetine) combined with oxybutynin significantly improved OSA severity when administered as a single dose before bed to a group of unselected OSA patients. Furthermore, neither combination significantly modified the key OSA endotypes at a group level, although there were large inter‐individual differences. Both combinations were able to lower the VRA but the effect magnitude was likely insufficient to provide a therapeutic benefit at the dose given. Contrary to previous trials using other noradrenergic agents (atomoxetine [Taranto‐Montemurro, Messineo, Sands, et al., 2019] or reboxetine [Lim et al., 2021]) the NSRI, milnacipran, did not have a significant effect on upper airway collapsibility or OSA severity when combined with an antimuscarinic. Conversely, we found that the more serotonergic potent Dul‐Oxy combination potentially improved airway collapsibility as shown by a shift to a greater proportion of respiratory events that were hypopnoeas. The results of this trial suggest that a potent and broad‐spectrum acting agent that exclusively increases norepinephrine is preferred for the antimuscarinic combination to provide significant efficacy in treating OSA.

## AUTHOR CONTRIBUTIONS

Conception and design: BE. Study coordination and data collection: LT, MA. Regulatory Management: LT, BE. Study physicians: AW, TC, SJ, GH. Data analysis and Interpretation of results: LT, SL, DM, MA, BE and colleagues. Initial manuscript writing: LT, BE. All authors interpreted data, edited the manuscript for important intellectual content, and approved the final draft.

## FUNDING INFORMATION

The current trial was sponsored by Apnimed Australia. A/Prof Edwards was supported by a Heart Foundation of Australia Future Leader Fellowship (101167). Dr. Joosten is supported by NHMRC Fellowship (1139745).

## CONFLICT OF INTEREST

Prof Hamilton has received equipment to support research from ResMed, Phillips Respironics and Air Liquide Healthcare. A/Prof Edwards receives grant support from Apnimed and NHMRC, and personal fees from Signifier Medical outside the current work. All other authors have no financial conflicts to disclose.

## Supporting information


Table S1

## Data Availability

In addition to the individual data provided in the manuscript, data that support the findings of this study may be available upon request to the sponsor at ltaranto@apnimed.com.
