# Supplementary material for: A single dose of noradrenergic/serotonergic reuptake inhibitors combined with an antimuscarinic does not improve obstructive sleep apnoea severity
Source: Physiol Rep. 2022 Aug 27;10(16):e15440. doi: 10.14814/phy2.15440 (PMC9419156; doi:10.14814/phy2.15440)
Supplement: Supplementary file 1 — Table S1 [file PHY2-10-e15440-s001.docx]

**A Single Dose of Dual Noradrenergic/Serotonergic Reuptake Inhibitors Combined with An Antimuscarinic Does Not Improve OSA Severity: A Pilot Study**

Luke DJ Thomson^1,2^, Shane A Landry^1,2^, Simon A Joosten^3,4,5^, Dwayne L Mann^1,6^, Ai-Ming Wong^4,5^, Tim Cheung^5^, Mulki Adams^1^, Caroline J Beatty^1,2^, Garun S Hamilton,^3,4,5^, Bradley A Edwards^1,2^

^1^Department of Physiology, School of Biomedical Sciences and Biomedical Discovery Institute, Monash University, Melbourne, VIC, Australia; ^2^Turner Institute for Brain and Mental Health, Monash University, Melbourne, VIC, Australia; ^3^School of Clinical Sciences, Monash University, Melbourne, VIC, Australia; ^4^Monash Lung, Sleep, Allergy and Immunology, Monash Health, Melbourne, VIC, Australia; ^5^Monash Partners – Epworth, Melbourne, VIC, Australia; ^6^School of Information Technology and Electrical Engineering, The University of Queensland, Brisbane QLD, Australia;

# **ONLINE DATA SUPPLEMENT**

Corresponding author: Bradley Edwards, PhD

Sleep and Circadian Medicine Laboratory,

Faculty of Medicine, Nursing and Health Sciences,

Monash University, Clayton, VIC 3168

Email: [bradley.edwards@monash.edu](mailto:bradley.edwards@monash.edu)

T: +61 3 99050187 | F: +61 3 99053948

**Supplementary results:**

**Table S1. Effects of treatment drugs on obstructive sleep apnea endotypes according to sleep state and body position**

|  | Placebo | Oxybutynin | Dul-Oxy | Mil-Oxy | *P v*alue |
| --- | --- | --- | --- | --- | --- |
| **NREM SLEEP (ALL POSITIONS):** | | | | | |
| Loop gain | 0.66 [0.59―0.85] | 0.61 [0.54―0.69] | 0.58 [0.52―0.75] | 0.65 [0.58―0.70] | 0.83 |
| VRA (%V_eupnea_) | 38 [23―49] | 29 [20―36] | **22 [15―36]*** | **27 [19―47]*** | **0.054** |
| ArTH (%V_eupnea_) | 121 [113―149] | 130 [105―161] | 114 [106―143] | 118 [110―134] | 0.83 |
| V_passive_ (%V_eupnea_) | 92 (± 5.9) | 91 (± 7.9) | 94 (± 5.4) | 93 (± 5.6) | 0.39 |
| V_active_ (%V_eupnea_) | 107 (± 14.5) | 99(± 27.3) | 103 (± 9.5) | 97 (± 17.7) | 0.22 |
| V_min_ (%V_eupnea_) | 62 (± 14.1) | 61 (± 14.2) | 65 (± 13.5) | 66 (± 9.4) | 0.25 |
| **ALL SLEEP STATES (ALL POSITIONS)** | | | | | |
| Loop gain | 0.59 [0.55―0.88] | 0.58 [0.52―0.64] | 0.58 [0.52―0.75] | 0.61 [0.54―0.71] | 0.41 |
| VRA (%V_eupnea_) | 35 [20―50] | 29 [20―42] | **22 [19―37]*** | **29 [17―42]*** | **0.03** |
| ArTH (%V_eupnea_) | 139 [117―162] | 139 [113―162] | 114 [108―148]* | 122 [118―144] | 0.09 |
| V_passive_ (%V_eupnea_) | 91 (± 7.1) | 90 (± 7.4) | 93 (± 6.0) | 93 (± 5.5) | 0.13 |
| V_active_ (%V_eupnea_) | 92 [63―116] | 97 [52―108] | 101 [95―107] | 87 [71―106] | 0.28 |
| V_min_ (%V_eupnea_) | 62 (± 14.1) | 60 (± 13.5) | 64 (± 13.0) | 65 (± 10.0) | 0.35 |
| **ALL SLEEP STATES (SUPINE)** | | | | | |
| Loop gain | 0.60 [0.55―0.75] | 0.61 [0.55―0.89] | 0.61 [0.55―0.73] | 0.63 [0.56―0.73] | 0.97 |
| VRA (%V_eupnea_) | 27 [19―66] | 32 [21―41] | 23 [14―31] | **24 [15―47]*** | **0.02** |
| ArTH (%V_eupnea_) | 143 [119―175] | 142 [122―165] | **117 [109―156]**** | 140 [121―150] | **0.02** |
| V_passive_ (%V_eupnea_) | 86 (± 12.0) | 84 (± 9.3) | 92 (± 6.8) | 87 (± 11.8) | 0.19 |
| V_active_ (%V_eupnea_) | 90 [65―114] | 81 [43―113] | 100 [91―106] | 79 [48―122] | 0.95 |
| V_min_ (%V_eupnea_) | 58 (± 14.7) | 56 (± 15.4) | 63 (± 16.5) | 58 (± 15.9) | 0.29 |

VRA = ventilatory response to arousal; Veupnea = eupneic ventilation; ArTH = arousal threshold; V_passive_ = ventilation when upper airway dilator muscles are hypotonic/passive; V_active_ = ventilation when upper airway dilator muscles are maximally activated; V_min_ = ventilation at lowest ventilatory drive; Dul-Oxy = duloxetine+oxybutynin; Mil-Oxy = milnacipran+oxybutynin. Data presented as means ± SD or median [interquartile range]. *n* = 9

* p ≤ 0.05; ** p ≤ 0.01; *** p ≤ 0.001
